# Collective Odor Source Estimation and Search in Time-Variant Airflow Environments Using Mobile Robots

**DOI:** 10.3390/s111110415

**Published:** 2011-11-02

**Authors:** Qing-Hao Meng, Wei-Xing Yang, Yang Wang, Ming Zeng

**Affiliations:** Institute of Robotics and Autonomous Systems, School of Electrical Engineering and Automation, Tianjin University, No. 92, Weijin Rd., Tianjin 300072, China; E-Mails: weixing@tju.edu.cn (W.-X.Y.); raynor63@163.com (Y.W.); zengming@tju.edu.cn (M.Z.)

**Keywords:** odor source localization, multi-robot, estimation, search, Bayesian rules, fuzzy inference, particle swarm optimization

## Abstract

This paper addresses the collective odor source localization (OSL) problem in a time-varying airflow environment using mobile robots. A novel OSL methodology which combines odor-source probability estimation and multiple robots’ search is proposed. The estimation phase consists of two steps: firstly, the separate probability-distribution map of odor source is estimated via Bayesian rules and fuzzy inference based on a single robot’s detection events; secondly, the separate maps estimated by different robots at different times are fused into a combined map by way of distance based superposition. The multi-robot search behaviors are coordinated via a particle swarm optimization algorithm, where the estimated odor-source probability distribution is used to express the fitness functions. In the process of OSL, the estimation phase provides the prior knowledge for the searching while the searching verifies the estimation results, and both phases are implemented iteratively. The results of simulations for large-scale advection–diffusion plume environments and experiments using real robots in an indoor airflow environment validate the feasibility and robustness of the proposed OSL method.

## 1. Introduction

The olfactory sense is crucial to the survival for many creatures, and has long played a fundamental role in human development and biosocial interaction. Electronic noses (e-noses), which are instruments designed to mimic the mammalian olfaction system, focus on identifying, classifying and quantifying the odor mixture—the fundamental function of animals’ smell sense. They are very useful for numerous applications in the food and pharmaceutical industry, in gaseous contamination monitoring, clinical diagnostics, contrabands inspection [1]. Besides the smell perception and discrimination, a number of life forms also use olfaction to trace odor cues for foraging, finding mates, exchanging information and evading predators [2]. Inspired by the odor tracing abilities of animals, in the early 1990s, researchers started trying to build mobile robots with similar olfaction abilities to replace trained animals [3–6]. It is expected that mobile robots developed with such olfaction abilities will play an increasing role in areas such as judging toxic or harmful gas leakage locations, searching for survivors in collapsed buildings, humanitarian de-mining and thwarting terrorist attacks.

Research into the use of one or more mobile robots equipped with odor/gas sensors and/or a wind sensor to search for odor/gas sources is called odor source localization (OSL) research [6,7]. OSL research can be classified into behavior-based methods and analytical-model-based methods [8].

The task of behavior-based OSL can be decomposed into three sub-procedures (namely, plume finding, plume traversal, and source declaration) according to Hayes *et al*. [7], or four sub-procedures (namely, finding a plume, tracing the plume, reacquiring the plume, and declaring the source) according to Li *et al*. [9]. During the initial phase, contact is made with an odor plume [7,9,10]. Once the plume is detected, the robot traces the odor/chemical toward its source. Most methods for this sub-procedure are biologically inspired, such as the gradient-following algorithms [10–12], the zigzagging algorithms [6,9,10,13], the upwind algorithms [7,14], and the SPIRAL algorithm (Searching Pollutant Iterative Rounding ALgorithm) [15]. To our knowledge, until now most research related to the OSL focuses on the plume tracing phase, that might be why the mobile-robot based OSL is also called chemical plume tracing (CPT) [16,17]. In the final phase, the robot locates the source [9,18,19].

Analytical-model based methods have also been proposed by several OSL researchers. The analytical-model based odor-source estimation could make up for the disadvantage of commonly used gas sensors with small detection range as well as the discrete distribution of plumes caused by turbulence. Ishida *et al*. [20] proposed a method to remotely locate a gas source based on a time-averaged gas distribution model [21]. Kowadlo and Russell [22] used a map of the robot’s environment, together with a naïve physics model of airflow, to predict the air movement pattern in a cluttered indoor environment with low ceiling and thinly populated by objects that affect airflow. The robot then used the airflow pattern to infer the probable location of the odor source. Farrell *et al*. [23] presented a plume mapping approach based on hidden Markov methods. Pang *et al*. [24] also proposed a source-likelihood mapping approach based on Bayesian inference method, where the source-likelihood map was propagated through time and updated in response to both detection and non-detection events. Li *et al*. [25] proposed a particle filter based algorithm to estimate the localization of the odor source in real time in a time-varying outdoor airflow environment.

Up to now, most OSL research work was implemented using a single robot. Compared with the single-robot search, multiple robots might have at least two advantages: the expected search time could be decreased; and multi-robot systems could provide a greater robustness against hardware failures. Hayes [7] proposed a spiral surge strategy for multiple robots OSL with real-robot hardware. Several fans were used to produce an artificial wind field. Lytridis and his colleagues [26] combined the biologically inspired chemotaxis strategy with biased random walking (BRW) strategy to form a chemo-BRW algorithm for multi-robot plume tracing with three BIRAW robots. A Gaussian-shaped odor field was created using a fan. The particle swarm optimization (PSO) algorithm was tested via computer simulation by Jatmiko [27] and Marques [28] using the plume models developed by Farrell [29] and Nielsen [30], respectively. Li and Meng [31] proposed a probability PSO (P-PSO) algorithm for multi-robot based OSL. Simulation results in ventilated indoor environments demonstrated the feasibility and advantage of the P-PSO algorithm. Spears and her colleagues [32] proposed a multi-robot CPT algorithm called fluxotaxis that follows the gradient of the chemical mass flux to locate a chemical source emitter. Ferri and his colleagues [15] used a biologically-inspired SPIRAL (Searching Pollutant Iterative Rounding ALgorithm) with MOMO (Multi-robot for Odor Monitoring) platform to localize a gas source in an indoor environment with no strong airflow. Meng *et al*. [33] applied an adapted ant colony optimization algorithm to multi-robot odor-plume tracing in indoor natural airflow environments, and real robot experiments demonstrated its feasibility.

Multi-robot based OSL has not been well studied and has mostly been restricted to simulated robots and simulation environments. To our knowledge, only a few publications [7,26,33] have discussed the OSL problem with multiple real robots, where the plumes in [7,26] were produced using fans instead of natural airflow. In indoor natural airflow environments the dispersion of odor molecules is dominated by turbulence. Here natural airflow means that the wind is not produced using fans. The natural wind direction in indoor environments often changes randomly and sometimes even by 180°. In addition, local concentration maxima caused by large eddies often exist in indoor environments, especially in corners.

A novel collective OSL strategy which combines multi-robot search with gas source probability estimation is proposed in this paper. The source probability estimation consists of two steps. Firstly, separate gas source probability map is estimated via Bayesian rules and fuzzy inference by using a single robot’s detection information; secondly, the distance and superposition methods are used to fuse separate source probability maps into one combined map. Multi-robot search is realized by a PSO algorithm, in which the local and global fitness functions are replaced by the estimated separate and combined gas source probability, respectively. The gas source probability estimation and multi-robot searching are implemented iteratively. The estimation phase exploits the detected information to guide multi-robot search, and multiple robots’ search can further verify the estimation result by updating their locations continuously. The proposed collective OSL strategy has been verified in both the simulated and real time-varying plume environments.

One of the main contributions of this manuscript is that a new OSL methodology which combines the estimation and searching is proposed. The previously published OSL work either only used behavior based searching methods or only used analytical based estimation methods, however, few research works related to OSL combining both the estimation and searching methods have been published. The behavior-based OSL searching without estimation has blindness, while the feedback of estimation based OSL is difficult to be obtained without robot searching. In the P-PSO algorithm proposed in our research, the estimation process exploits the detected information to guide the multi-robot search, while the multi-robot search coordinated via PSO method updates the estimation result by exploring more areas, thus a better OSL performance could be achieved.

The remainder of this paper is organized as follows. The characteristics of an advection-diffusion plume are analyzed in Section 2. Section 3 presents the framework of the collective OSL strategy. The realization of separate and combined gas-source probability estimation is explained in Section 4. Section 5 introduces PSO-based collective gas-source search strategy using the estimated source probability as the fitness function. The simulation and multiple real-robot experiments for collective OSL and search are given in Sections 6 and 7, respectively. Finally, conclusions are summarized.

## 2. Characteristic of Advection-Diffusion Plume

The transport of a gas in the air is influenced by advection, turbulent diffusion and molecular diffusion. The effect of advection is that the gas is transferred by the time-averaged flow movement; the effect of turbulent diffusion is that the gas diffuses by turbulent kinetics; the molecular diffusion is caused by molecular motion. The speed of turbulent diffusion is much faster than that of molecular diffusion. For example, in the air the difference is about 10^5^–10^6^ times, therefore the molecular diffusion in turbulence could be neglected [34]. Because the height of gas sensor equipped on the robot is usually fixed, the equation that describes advection-diffusion of a puff is formulated for the case of sensing a 2-dimension plane as follows [29,35]:

(1) $$\dot{X}(t)=U(X,t)+N(t)$$

where **X**(*t*) = (*x*(*t*),*y*(*t*)) is the coordinate of the puff at time *t*; **U** = (*u_x_*, *u_y_*) is the velocity of advection; **N** = (*v_x_*, *v_y_*) represents the turbulent diffusion, which could be expressed by a quasi-Gaussian random process [36] with expectation (0,0) and variance ( $\omega_{x}^{2}$, $\omega_{y}^{2}$).

Without loss of generality, suppose the wind direction is along the x axis, *i.e*., the movement is only dominated by advection along the x axis direction, and the random process of Gaussian distribution is only considered in y axis. A puff was released at the time *t* = 0 from the origin, and the displacement of the puff in the y axis direction is given by:

$$y(t)=\int_{0}^{t}v_{y}(t^{\prime })dt^{\prime }$$

Since *v_y_* fits the Gaussian distribution, the ensemble average displacement of large puffs is $\bar{y(t)}=0$ and the variance $\bar{y{\left(t\right)}^{2}}$ [36] can be expressed as follows:

(2-a) $$\bar{y{\left(t\right)}^{2}}=2\omega_{y}^{2}\int_{0}^{t}(t-\tau )\text{exp}\left(-\frac{\tau }{T_{L}}\right)d\tau$$

For a short period of time, Equation (2-a) can be rewritten as follows:

(2-b) $$\bar{y{\left(t\right)}^{2}}\approx \omega_{y}^{2}t^{2}$$

**Figure 1.**
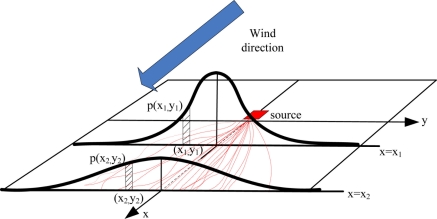
The schematic diagram of probability density distribution of puffs.

For a long period of time, Equation (2-a) can be rewritten as follows:

(2-c) $$\bar{y{\left(t\right)}^{2}}\approx 2\omega_{y}^{2}T_{L}t$$

where *T_L_* represents the Lagrange time scalar. From Equations (2-b) and (2-c) it can be found that the variance in the y axis direction increases gradually with time. In a short time, $\bar{y{\left(t\right)}^{2}}$ is directly proportional to the square of time, while in a long time, $\bar{y{\left(t\right)}^{2}}$ is proportional to the time. The movements of puffs are illustrated in Figure 1. The red dashed-lines denote the trajectories of puffs in x−y plane. In homogeneous isotropic turbulence, the probability distribution of puffs in cross section of the x axis fits the Gaussian distribution [37]. *p*(*x,y*) denotes the probability density function (PDF) of puffs in cross section of the x axis; the puffs arrive at the lines *x* = *x*_1_ and *x* = *x*_2_ at time *t*_1_ and *t*_2_, respectively, so $\bar{y{\left(t_{1}\right)}^{2}}$ and $\bar{y{\left(t_{2}\right)}^{2}}$ denote the variances of the PDF along the lines *x* = *x*_1_ and *x* = *x*_2_, respectively. In Figure 1, *t*_2_ > *t*_1_, $\bar{y_{2}{\left(t\right)}^{2}}>\bar{y_{1}{\left(t\right)}^{2}}$ according to Equation (2-b), so in the same abscissa *y*_1_ = *y*_2_, *p*(*x*_1_,*y*_1_) > *p*(*x*_2_,*y*_2_). The characteristic of advection-diffusion is fundamental to the collective gas-source probability estimation presented in Section 4.

## 3. Framework of the Collective OSL Strategy

**Figure 2.**
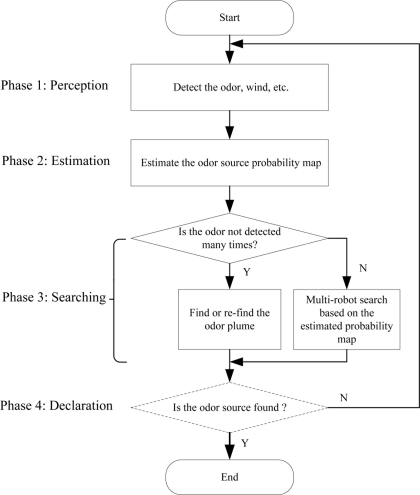
The flowchart of the proposed gas source localization strategy.

In the proposed collective OSL process, the gas-source probability estimation and multi-robot search are implemented iteratively. The estimation is used for guiding robots’ search, and multiple robots’ search can further verify the estimation result by updating their locations continuously. The proposed methodology includes the following four phases. The flowchart is illustrated in Figure 2.

*Phase 1*: Perception, *i.e*., detection of gas and airflow data using onboard sensors.

*Phase 2*: Estimation, *i.e*., prediction of gas source position by fusing the detected gas and airflow information. The gas source position prediction consists of two steps, the first is separate prediction using Bayesian rules and fuzzy inference based on single robot’s detection events, the second is the estimation of the combined probability map using the distance based superposition method.

*Phase 3*: Multi-robot search, a particle swarm optimization (PSO) algorithm is used to coordinate multi-robot based collective search for the gas source. The PSO uses the estimated separate and conbined gas source probabilities instead of real values (gas concentration, for example) as the local and global fitness functions, respectively. Here we call it the Probability-fitness-function based Particle Swarm Optimization (P-PSO) algorithm. If all the searchers have not detected the gas after many tries, a simple heuristic finding/re-finding plume algorithm is used; otherwise, the robots move according to the P-PSO searching algorithm based on the gas-source probability estimation.

*Phase 4:* Declaration, *i.e*., identification of the gas source. If the source is not successfully declared, the algorithm will return to the phase 1.

The algorithm proposed by Li *et al*. [18] could be used for the declaration phase (dashed-line frame in Figure 2), but in our experiments, the multiple robots were stopped manually when all the robots approached the real source and converged to a specified area.

## 4. Gas Source Probability Estimation

The gas source probability estimation consists of two steps, the first is separate estimation based on single robot’s detection events by using Bayesian rules and fuzzy inference (see Section 4.1), the second is fusing the separate estimation to form a combined probability map by using the distance based superposition method (see Section 4.2).

### 4.1. Separate Gas Source Probability Estimation

Because the accurate turbulent model is hard to set up, the posterior probability of gas source could not be obtained from it. The separate gas source probability estimated using Bayesian rule is expressed as follows:

(3-a) $$p(m|z)=\frac{p(m|z,\Delta )p(\Delta |z)}{p(\Delta |z,m)}$$

where *p*(*m*|*z*) denotes the posterior probability of the area *m* being the gas source given the detection event of the robot’s sensors. The sensor detection event *z* denotes the detection of wind and gas. *m*, the smallest area unit of gas source probability estimation, denotes the grid in searching area; Δ denotes a small square connected domain, and *m* ⊂ Δ; *p*(*m*|*z*,Δ) expresses the probability of the unit *m* being the gas source given the detection event of the sensors and the source being in the domain Δ. *p*(Δ|*z*) stands for the probability of the gas source being located in the domain Δ given the sensor detection event.

∵ *m* ⊂ Δ, ∴ *p*(Δ|*z*,*m*) = 1. So Equation (3-a) can be rewritten as follows:

(3-b) p(m|z)=p(Δ|z)p(m|z,Δ)

**Figure 3.**
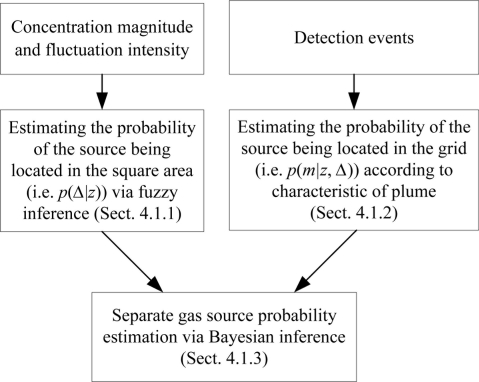
The flowchart of the separate gas source probability estimation.

The estimation process of the probabilities *p*(Δ|*z*) and *p*(*m*|*z*,Δ) is presented in Sections 4.1.1 and 4.1.2, respectively. The flowchart of the separate gas-source probability estimation is shown in Figure 3.

#### 4.1.1. Estimation of *p*(Δ|*z*)

*p*(Δ|*z*) is estimated using fuzzy inference by combining the concentration magnitude and fluctuation intensity. The gas plume itself contains the information about source location, and the fluctuation intensity can be used to express the gas variation [15]. Here the fluctuation intensity is set to be the number of wave peaks whose values are bigger than the average. The inputs of the fuzzy inference are the gas concentration and its variation, and the output is the estimated probability *p*(Δ|*z*). The concentration information is calculated by sampling many times and then averaging. Both the inputs and output of the fuzzy inference are divided into five fuzzy subsets: SMALL, MIDDEL-SMALL, MIDDLE, MIDDLE-BIG and BIG. The central idea of the fuzzy reasoning rules is that, the less concentration and fluctuation intensity, the less probability of gas source being in the area Δ, and *vice versa* [15]. To adapt to the change of environment, especially under the condition of the unknown gas source concentration, the universe of the fuzzy sets is variable instead of fixed. The two fuzzy input universe ranges are set to 2*C*_max_ and 2*I*_max_, respectively. *C*_max_ and *I*_max_ are the maximal gas concentration and fluctuation intensity detected until now, respectively.

#### 4.1.2. Estimation of *p*(*m*|*z*,Δ)

According to the dynamic characteristics of gas plume described in Section 2, the square area Δ centered on the gas and wind sensors is determined as follows:

(4) $$r_{t}=r_{0}\left(1-p\left(\Delta |z_{t}\right)\right)$$

where *r_t_* denotes the side length of the area Δ at the time *t*; the initial side length *r*_0_ is set to 10 m in simulations and 2 m in experiments; *p*(Δ|*z_t_*) denotes the probability of gas source located in Δ given the detection value *z_t_*. The value of *p*(Δ|*z_t_*) is obtained by the fuzzy inference (see Section 4.1.1). The reason that *r_t_* is set to be variable is that, the estimation area is narrowed when the detected concentration increases, so that the robot is prone to search locally near the location of high concentration and the exploitation performance of the robots increases; in the position of low concentration, the enlargement of estimation area could broaden the search area of the robot, and the exploration performance of the robots gets enhanced.

Suppose *c_ref_* denotes the detection threshold of gas sensors (*c_ref_* = 50 ppm in our experiments). Let *c_t_* denote the average gas concentration when the robot detects gas in the time period [*t*, *t + λt*], where *λt* is the sampling period of sensors. Let *z_t_* denote the detection event *z* happening in the time period [*t*, *t + λt*]. *z_t_* has two forms: *z_t,In_* (*i.e*., *c_t_* ≥ *c_ref_*) and $\bar{z_{t}}$ (*i.e*., *c_t_* < *c_ref_*). $\bar{z_{t}}$ also includes two forms: $\bar{z_{t,\mathrm{Edge}}}$ and $\bar{z_{t,\mathrm{Out}}}$. The detection event *z_t,In_* means the robot is in the plume; while the detection event $\bar{z_{t,\mathrm{Edge}}}$ and $\bar{z_{t,\mathrm{Out}}}$ denote the robot is on the edge and outside of the plume, respectively. Here we distinguish these two situations by setting a time threshold *T_plume_* (10 s in our experiments). If the time of not detecting gas is greater than *T_plume_*, the robot is considered to be outside of the plume; otherwise the robot is considered to be on the edge of the plume. Under the three kinds of detection events, *i.e*., *z_t,In_*, $\bar{z_{t,\mathrm{Edge}}}$ and $\bar{z_{t,\mathrm{Out}}}$, the probability estimation of gas source is constructed through the wind direction information.

**Figure 4.**
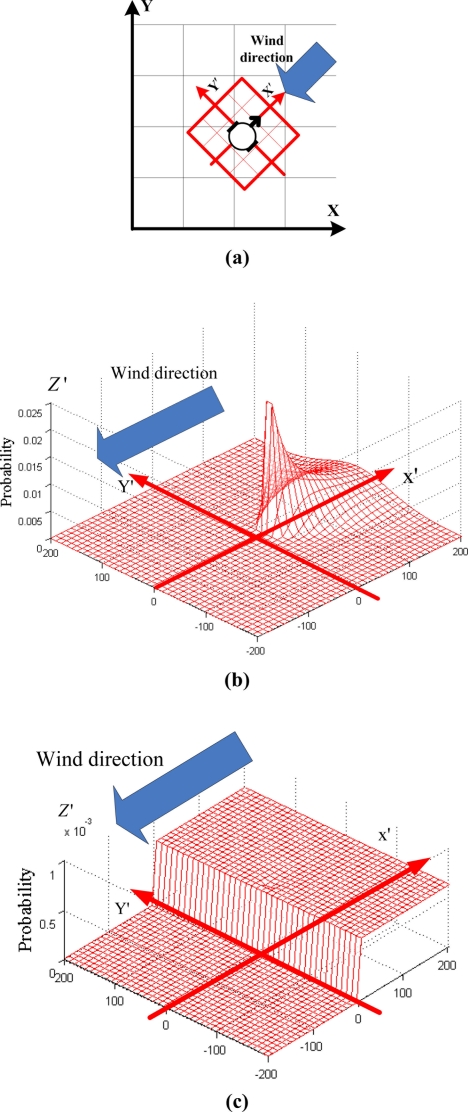
**(a)** The grids in the robot and world coordinate systems; **(b)** Separate gas source probability distribution map (the gas is detected); **(c)** Separate gas source probability distribution map (the gas is undetected).

As Figure 4(a) shows, we take the location of the robot as the origin of coordinate, and the directions parallel and perpendicular to the upwind direction as the lateral and vertical axes (see X′ and Y′), respectively. The square area Δ (see the square marked with red thick line) is divided into grids *m*_*x′y′*_ with the central coordinate (*x′*,*y′*) in (robot’s) local coordinate system X′−Y′. Let *m_xy_* represent the grid *m* with the central coordinate (*x,y*) in global (world) coordinate system. The global coordinate and local coordinate systems can be transformed by the equation *f*(*m*_*x′y′*_) = *m_xy_*.

It is supposed that each detected filament travels directly from the gas source to the sensor in the estimated area. The separate posterior probability *p*(*m*_*x′y′*_|*z_t_*,Δ) of grid *m*_*x′y′*_ being gas source in the area Δ by the detection event *z_t_* can be calculated according to the Bayesian rules. The movement time of the detected puff is previously unknown, so in square area Δ the moving time of the detected puff is supposed from 0 to *t_M_*, where *t_M_* represents the maximum moving time of the detected puff in Δ, its value can be calculated as:

(5) $$t_{M}=\frac{r_{t}}{2u_{x^{\prime }}}$$

where *u*_*x′*_ denotes the wind magnitude. When the event *z_t,In_* happens and the movement time of the detected puff is equal to $\frac{x^{\prime }}{u_{x^{\prime }}}$, *p*(*m*_*x′y′*_|*z_t_*,Δ) is calculated using Equation (6); when the movement time of the detected puff is not $\frac{x^{\prime }}{u_{x^{\prime }}}$, *p*(*m*_*x′y′*_|*z_t_*,Δ) is equal to 0.

(6) $$p\left(m_{x^{\prime }y^{\prime }}|z_{t,\mathrm{In}},\Delta \right)=\{\begin{array}{cc} \frac{p\left(m_{x^{\prime }y\prime }\right)p\left(z_{t,\mathrm{In}},\Delta |m_{x^{\prime }y\prime }\right)}{p\left(z_{t,\mathrm{In}},\Delta \right)}=\frac{p\left(m_{x^{\prime }y^{\prime }}\right)p\left(z_{t,\mathrm{In}}|m_{x^{\prime }y^{\prime }}\right)}{\sum_{m_{x^{\prime }y\prime }\in \Delta }p\left(m_{x^{\prime }y\prime }\right)p\left(z_{t,\mathrm{In}}|m_{x^{\prime }y\prime }\right)}, & \mathrm{if}\;x^{\prime }>\frac{d_{\mathrm{robot}}}{2}\\ \varepsilon , & \mathrm{if}\;x^{\prime }\leq \frac{d_{\mathrm{robot}}}{2} \end{array}$$

where *p(m*_*x′y′*_) denotes the separate prior probability that the gas source is in the grid *m*_*x′y′*_. Considering the volume of robot, the initial abscissa in the estimation area should be more than half of the robot side length *d_robot_*. In the downwind field, the posterior probability of the grid being gas source is set to be a small constant ɛ. In the upwind field, the conditional probability *p(z*_*t,In*_|*m*_*x′y′*_) is represented as follows [37] (see also Figure 1).

(7) $$p\left(z_{t,\mathrm{In}}|m_{x^{\prime }y\prime }\right)=\frac{1}{\sqrt{2\pi \sigma_{y^{\prime }}^{2}}}\text{exp}\left(-\frac{d^{2}}{2\sigma_{y^{\prime }}^{2}}\right)=\frac{1}{\sqrt{2\pi \sigma_{y^{\prime }}^{2}}}\text{exp}\left(-\frac{{y^{\prime }}^{2}}{2\sigma_{y^{\prime }}^{2}}\right)$$

where *y′* represents the ordinate of central position of the grid *m*_*x′y′*_; *d* denotes the distance between the central point of the grid *m*_*x′y′*_ and the square’s centerline being parallel with the wind direction (see Figure 4); $\sigma_{y^{\prime }}^{2}$ represents the variance of the probability distribution of the puffs in the vertical direction of the wind when the puffs diffuse from the grid *m*_*x′y′*_ to the position of the sensor. Here the prior probability *p*(*m*_*x′y′*_) in Δ is set to be equal in separate gas source probability estimation, so it can be deleted. Substituting Equation (7) into Equation (6), we get:

(8) $$p\left(m_{x^{\prime }y\prime }|z_{t,\mathrm{In}},\Delta \right)=\frac{\frac{1}{\sqrt{2\pi }\sigma_{y^{\prime }}}\cdot \text{exp}\left(-\frac{{y^{\prime }}^{2}}{2\sigma_{y^{\prime }}^{2}}\right)}{\sum_{m_{x^{\prime }y\prime }\in \Delta }\frac{1}{\sqrt{2\pi }\sigma_{y^{\prime }}}\cdot \text{exp}\left(-\frac{{y^{\prime }}^{2}}{2\sigma_{y^{\prime }}^{2}}\right)}=\frac{\frac{1}{\sigma_{y^{\prime }}}\cdot \text{exp}\left(-\frac{{y^{\prime }}^{2}}{2\sigma_{y^{\prime }}^{2}}\right)}{\sum_{m_{x^{\prime }y\prime }\in \Delta }\frac{1}{\sigma_{y^{\prime }}}\text{exp}\left(-\frac{{y^{\prime }}^{2}}{2\sigma_{y^{\prime }}^{2}}\right)},x^{\prime }>\frac{d_{\mathrm{robot}}}{2}$$

Let *ω_y′_* express the standard deviation of random process of turbulence in Y′ direction, then $\sigma_{y^{\prime }}=t_{x^{\prime }}\cdot \omega_{y^{\prime }}=\frac{x^{\prime }}{u_{x^{\prime }}}\cdot \omega_{y^{\prime }}$. [see Equation (2-b)], where *t_x_*_′_ denotes the moving time of the puffs from the grid *m*_*x′y′*_ to the position of the sensor. Therefore Equation (8) can be rewritten as follows:

(9) $$p\left(m_{x^{\prime }y\prime }|z_{t,\mathrm{In}},\Delta \right)=\frac{\frac{1}{\sigma_{y^{\prime }}}\cdot \text{exp}\left(-\frac{{y^{\prime }}^{2}}{2\sigma_{y^{\prime }}^{2}}\right)}{\sum_{m_{x^{\prime }y\prime }\in \Delta }\frac{1}{\sigma_{y^{\prime }}}\text{exp}\left(-\frac{{y^{\prime }}^{2}}{2\sigma_{y^{\prime }}^{2}}\right)}=\frac{\frac{1}{x^{\prime }}\cdot \text{exp}\left(-\frac{{y^{\prime }}^{2}}{2\sigma_{y^{\prime }}^{2}}\right)}{\sum_{m_{x^{\prime }y\prime }\in \Delta }\frac{1}{x^{\prime }}\text{exp}\left(-\frac{{y^{\prime }}^{2}}{2\sigma_{y^{\prime }}^{2}}\right)},x^{\prime }>\frac{d_{\mathrm{robot}}}{2}$$

The separate probability map in square area Δ with the supposed moving time of the detected puff from 0 to *t_M_* is shown in Figure 4(b), where the value in Z′ axis shows the probability details.

When the robot does not detect the gas, the posterior probability distribution is represented as follows:

(10-a) $$p\left(m_{x^{\prime }y\prime }|{\bar{z}}_{t},\Delta \right)=\frac{p\left(m_{x^{\prime }y\prime }\right)p\left({\bar{z}}_{t,}\Delta |m_{x^{\prime }y\prime }\right)}{p\left({\bar{z}}_{t},\Delta \right)}=\frac{p\left(m_{x^{\prime }y\prime }\right)p\left({\bar{z}}_{t}|m_{x^{\prime }y\prime }\right)}{\sum_{m_{x^{\prime }y\prime }\in \Delta }p\left(m_{x^{\prime }y\prime }\right)p\left({\bar{z}}_{t}|m_{x^{\prime }y\prime }\right)}=\frac{p\left({\bar{z}}_{t}|m_{x^{\prime }y\prime }\right)}{\sum_{m_{x^{\prime }y\prime }\in \Delta }p\left({\bar{z}}_{t}|m_{x^{\prime }y\prime }\right)}=\frac{1-p\left(z_{t}|m_{x^{\prime }y\prime }\right)}{\sum_{m_{x^{\prime }y\prime }\in \Delta }\left(1-p\left(z_{t}|m_{x^{\prime }y\prime }\right)\right)}$$

(10-b) $$p\left(m_{x^{\prime }y\prime }|{\bar{z}}_{t},\Delta \right)=\frac{1-\frac{1}{\sqrt{2\pi \sigma_{y^{\prime }}}}\cdot \text{exp}\left(-\frac{{y^{\prime }}^{2}}{2\sigma_{y^{\prime }}^{2}}\right)}{\sum_{m_{x^{\prime }y\prime }\in \Delta }\left(1-\frac{1}{\sqrt{2\pi \sigma_{y^{\prime }}}}\cdot \text{exp}\left(-\frac{{y^{\prime }}^{2}}{2\sigma_{y^{\prime }}^{2}}\right)\right)}$$

When $\bar{z_{t,\mathrm{Edge}}}$ and $\bar{z_{t,\mathrm{Out}}}$ happen, the estimation probability distribution map is shown in Figure 4(c). We can obtain an approximate template of separate probability distribution of gas source in the area Δ by detecting the gas and wind (*cf.* [Figure 4(b,c)]). The purpose of estimating the separate map is that the discrete detection point can be converted into a continuous probability distribution field, which makes up the disadvantage of gas sensor with small detection range as well as the discrete distribution of plume caused by turbulence.

#### 4.1.3. Separate Gas Source Probability Estimation

When the detection event *z_t,In_* happens, the separate gas source probability *p(m*_*x′y′*_|*z_t,In_*) can be represented as follows on the basis of Equation (3-b),

(11) $$p\left(m_{x^{\prime }y\prime }|z_{t,\mathrm{In}}\right)=p\left(m_{x^{\prime }y^{\prime }}|z_{t,\mathrm{In}},\Delta \right)p\left(\Delta |z_{t,\mathrm{In}}\right)$$

When the detection events $\bar{z_{t,\mathrm{Edge}}}$ and $\bar{z_{t,\mathrm{Out}}}$ happen, $p\left(m_{x^{\prime }y^{\prime }}|\bar{z_{t,\mathrm{Edge}}}\right)$ and $p\left(m_{x^{\prime }y^{\prime }}|\bar{z_{t,\mathrm{Out}}}\right)$ are calculated using Equations (12) and (13), respectively:

(12) $$p\left(m_{x\prime y^{\prime }}|\bar{z_{t,\mathrm{Edge}}}\right)=\xi_{1}\cdot p\left(\Delta |z_{t-1,\mathrm{In}}\right)\cdot p\left(m_{x^{\prime }y\prime }|{\bar{z}}_{t,}\Delta \right)$$

(13) $$p\left(m_{x\prime y^{\prime }}|\bar{z_{t.,\mathrm{Out}}}\right)=\xi_{2}\cdot p\left(m_{x\prime y^{\prime }}|{\bar{z}}_{t,}\Delta \right)$$

where the symbols ξ_1_ and ξ_2_ are two constants with the range of (0, 1). For the detection events $\bar{z_{t,\mathrm{Edge}}}$ and $\bar{z_{t,\mathrm{Out}}}$, the fuzzy inference cannot be used to predict $p\left(\Delta |\bar{z_{t,\mathrm{Edge}}}\right)$ and $p\left(\Delta |\bar{z_{t,\mathrm{Out}}}\right)$. In Equation (12) ξ_1_ · *p*(Δ|*z*_*t*−1,*In*_) is used to approximate the value of $p\left(\Delta |\bar{z_{t,\mathrm{Edge}}}\right)$ (in our experiments, ξ_1_ was set to 0.8). In Equation (13), the probability $p\left(\Delta |\bar{z_{t,\mathrm{Out}}}\right)$ is set to be the small constant ξ_2_ (in our experiments, ξ_2_ was set to 0.2). The value of *p*(*m*_*x′y′*_|*z_t_*) in the local coordinate system X′−Y′ is transformed to the *p*(*m*_*xy*_*|z*_*t*_) in the world coordinate system X−Y by the transform equation *f*(*m*_*x′y′*_) = *m_xy_*.

### 4.2. Combined Gas Source Probability Estimation

The purpose of estimating combined gas source probability map is to guide the subsequent search of robots. To make the estimation results more reliable, all the separate probability maps from different spaces and different time are merged into one combined gas source probability map.

When *N* robots have detected the gas and/or wind information in the time period [*t*, *t* + *λt*], all the separate gas source probability distribution maps are integrated into a combined gas source probability map as follows.:

(14) $$p\left(m_{\mathrm{xy}}|Z_{t}\right)=\frac{\sum_{i=1}^{N}\rho_{i}\cdot p\left(m_{\mathrm{xy}}|z_{i,t}\right)}{N},\;\;Z_{t}=\left\{z_{1,t},z_{2,t},\ldots ,z_{N,t}\right\},\;\;\rho_{i}=\frac{\frac{1}{\left\|x_{x,y}-x_{i}\right\|}}{\sum_{j=1}^{N}\frac{1}{\left\|x_{x,y}-x_{j}\right\|}}$$

where **x**_*x*,*y*_ denotes the central coordinates of the grid *m_xy_*; **x***_i_* denotes the location of the *i*-th robot; *z*_*i*,*t*_ (*i* = 1,2,…,*N*) denotes the detection event by the robot *i* in the time period [*t*, *t* + *λt*]. Equation (14) shows that the closer the detected point is to the estimated grid, the bigger weight the estimated gas source probability has in the process of fusion.

Finally the combined gas source probability map is calculated by superposing the maps at different sampling times:

(15) $$p\left(m_{\mathrm{xy}}|Z_{1:t}\right)=\delta \cdot p\left(m_{\mathrm{xy}}|Z_{1:t-1}\right)+(1-\delta )p\left(m_{\mathrm{xy}}|Z_{t}\right),\;\;Z_{1:t}=\left\{Z_{1},Z_{2},\ldots ,Z_{t}\right\}$$

where *δ* denotes the decay coefficient of the history information. Through the iteration in time, an average distribution is obtained.

## 5. Multi-Robot Search

A particle swarm optimization (PSO) algorithm is used to coordinate multiple robots search for the gas source. The PSO uses the estimated separate and combined gas source probabilities instead of real values (gas concentration, for example) as the local and global fitness functions, respectively. Here we call it the Probability-fitness-function based Particle Swarm Optimization (P-PSO) algorithm.

If all the searchers have not detected the gas after many attempts, robots move toward different directions to re-find the plume; otherwise, the robots move according to the searching algorithm based on the result of estimation. The basic formula of standard PSO [38] can be expressed as follows:

(16) $$V_{i}(t)=wV_{i}(t-1)+c_{1}r_{1}\left({\mathrm{pos}}_{i}(t-1)-X_{i}(t-1)\right)+c_{2}r_{2}\left({\mathrm{pos}}_{g}(t-1)-X_{i}(t-1)\right)$$

(17) $$X_{i}(t)=X_{i}(t-1)+V_{i}(t)$$

where *c*_1_ and *c*_2_ are two constants; *w* is the inertial weight; *r*_1_ and *r*_2_ denote two random numbers; **X** *_i_*(*t*) and **V***_i_*(*t*) stand for the coordinate and velocity of the *i*-th robot at the time *t*, respectively; **pos***_i_*(*t*) and **pos***_g_*(*t*) represent the position of the optimal fitness function of the *i*-th robot and the position of global optimal fitness function, respectively.

In the P-PSO, **pos***_i_*(*t*) is the grid of the maximal gas-source posterior probability expectation of the *i*-th robot till the time *t*, and **pos***_g_*(*t*) represents the grid with the maximum combined gas-source posterior probability up to the time *t*. Here the estimated maximum probability of gas source in the local area is not adopted in P-PSO. The main reason is that we should consider the moving time of the detected puff. Based on the hypothesis of different moving times of the detected puff, we can get the gas probability distribution map shown in Figure 4(b,c) in a small area range. However, the accurate moving time is unknown in advance. Since the expected grid of probability distribution in small area Δ can denote the average position of the estimated gas source by this detection. The calculation formula of the expectation is expressed as follows:

(18) $$E_{i,t}{\left(m_{\mathrm{xy}}\right)}_{(x,y)\in \Delta }=\left(\frac{\sum_{x\in \Delta }x\cdot p\left(m_{\mathrm{xy}}|z_{i,t}\right)}{\sum_{x\in \Delta }x},\frac{\sum_{y\in \Delta }y\cdot p\left(m_{\mathrm{xy}}|z_{i,t}\right)}{\sum_{y\in \Delta }y}\right)$$

*p*(*m_xy_*|*z_i,t_*), which can be calculated through Equations (11)–(13) and coordinate transformation, represents the estimated probability of the gas source being in *m_xy_* by the detection event of the robot *i* at the time *t*.

In the proposed P-PSO, **pos***_i_*(*t*) and **pos***_g_*(*t*) are represented as follows:

(19-a) $${\mathrm{pos}}_{g}(t)=\underset{m_{\mathrm{xy}}}{\text{arg}}\mathrm{Max}\left(p\left(m_{\mathrm{xy}}|Z_{1:t}\right)\right)$$

(19-b) $${\mathrm{pos}}_{i}(t)=\underset{m_{\mathrm{xy}}}{\text{arg}}\mathrm{Max}\left(E_{i,t}{\left(m_{\mathrm{xy}}\right)}_{(x,y)\in \Delta }\right)$$

The necessity of coordinating multi-robot to search gas source by P-PSO includes two aspects. First, the proposed P-PSO uses the estimation probability distribution as a clue for re-finding the plume, thus it could reduce the probability of losing the plume. Second, the real gas concentration fluctuates violently, but the probability distribution changes slowly, so the probability distribution instead of real concentration is adopted as the fitness function.

## 6. Simulation Results and Analysis

### 6.1. Basic Simulation Assumptions

The size of the robot is negligible compared with the large scale of the search space (100 m × 100 m). It is assumed that each robot is equipped with one gas sensor and one wind sensor. The gas sensor has relatively quick response and recovery (further details are presented in Section 6.3). The wind sensor measures wind speeds from 0 to 10 m/s and wind directions from 0° to 359°. Zero-mean Gaussian noise is added to the output of the wind sensor, and the variances of the wind speed and direction are set to 0.05 m/s and 1°, respectively. The sampling frequency of the gas concentration and wind sensors is 10 Hz. In view of the influence of the recovery and response time of metal oxide semiconductor (MOS) sensors, the motion mode of “run-stop-run-stop” is adopted here. Each robot stops at one location for 5 s to collect the gas and wind information, and then the robot runs for 1 s again according to the velocity and direction calculated by the algorithm. Each robot knows its current location and moves in a speed ranging from 0.2 m/s to 0.8 m/s. The initial and largest side length of Δ is set to be 10 m. The smallest side length of Δ is 2 m. Gas concentration and wind information data recorded by the robots are sent to a workstation via wireless communication. The motion mode of each robot is planned by the algorithm running in the workstation.

### 6.2. Time-Variant Large-Scale Advection—Diffusion Plume Model

In Farrell’s model [29], a sequence of gas puffs is released at the source location, and each puff is composed of *n* filaments. The motion velocity of each filament is divided into three components: $\vec{V_{d}}$, $\vec{V_{m}}$, and $\vec{V_{a}}$, where each component is implemented by a distinct process. This decomposition of the velocity spectrum can be interpreted theoretically in terms of eddy scales. The effect of the smallest eddies (*i.e*., slow growth of the filaments) of the wind fluid flow process (modeled by $\vec{V_{d}}$) is implemented as an increase in filament size and a change in shape. The term $\vec{V_{a}}$ represents the portion of the wind process with characteristic length much larger than the filaments. This portion of the wind process transports each filament as a body; therefore, the term $\vec{V_{a}}$ represents advection. The advection portion of the velocity is represented as a continuous (in time and space) but temporally and spatially varying function, so that a sequence of filaments released at the source will result in a sinuous trail of filaments leaving the source. The term $\vec{V_{m}}$ represents the intermediate range of scales that transports (*i.e*., stirs) the filaments within the body of the plume.

The advection–diffusion model is composed of a large number of advected and dispersed filaments. Given the large number of filaments, the overall instantaneous concentration at **x** = (*x,y,z*) is the sum of the concentrations at that location contributed by each filament [29]:

(20) $$C(x,t)=\sum_{i=1}^{N}C_{i}(x,t)\frac{\mathrm{molecules}}{{\mathrm{cm}}^{3}}$$

where *N* is the number of filaments currently being simulated. The concentration at location **x** caused by the *i*th filament is modeled as [29]:

(21) $$C_{i}(x,t)=\frac{Q}{\sqrt{8\pi^{3}}R_{i}^{3}(t)}\text{exp}\left(\frac{-r_{i}^{2}(t)}{R_{i}^{2}(t)}\right)\frac{\mathrm{molecules}}{{\mathrm{cm}}^{3}\mathrm{filament}}$$

(22) $$r_{i}(t)=\left\|x-p_{i}(t)\right\|\mathrm{cm}$$

where *Q* represents the amount of gas released (*i.e*., molecules per filament), *R_i_* is a parameter controlling the size of the *i*th filament, and **p***_i_* (*t*) is the spatial extent of the *i*th filament.

### 6.3. Gas Sensor Model

**Figure 5.**
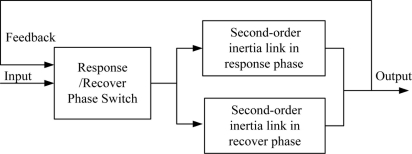
The MOS sensor model.

MOS sensors are widely used for chemical plume tracing because of their low cost and small size. To simulate the real response and recovery characteristics of MOS sensors, a second-order sensor model is built here, with the response and recovery phases of the sensors both regarded as second-order inertia links. The two phases have different time constants, and therefore their design parameters are different. The left block in Figure 5 represents the switch module for the two phases, and the right two blocks represent inertia links of the two phases. When the output is greater than the input, the recovery phase is chosen; otherwise, the response phase is chosen. Without considering the noise, the transfer functions for the second-order inertia links in the response and recovery phases are expressed as Equations (23) and (24), respectively:

(23) $$\frac{y_{\mathrm{res}}(s)}{x(s)}=\frac{1}{\left(1+A_{\mathrm{res}}s\right)\left(1+B_{\mathrm{res}}s\right)}$$

(24) $$\frac{y_{\mathrm{rec}}(s)}{x(s)}=\frac{1}{\left(1+A_{\mathrm{rec}}s\right)\left(1+B_{\mathrm{rec}}s\right)}$$

where *x*(*s*) is the input and *y_res_*(*s*) and *y_rec_*(*s*) are the outputs of the sensor in the response and recovery phases, respectively.

In our simulations, the discrete sensor models [Equations (25) and (26)] with additive Gaussian noise are used:

(25) $$\{\begin{array}{l} y_{\mathrm{res}}(k)=a_{\mathrm{res}}x(k)+b_{\mathrm{res}}y_{\mathrm{res}}(k-1)-c_{\mathrm{res}}y_{\mathrm{res}}(k-2)\\ \;\;\;\;\;\;\;\;\;\;\;\;\;\;\;\;\;\;+n_{\mathrm{res}}(k)\\ a_{\mathrm{res}}+b_{\mathrm{res}}-c_{\mathrm{res}}=1 \end{array}$$

(26) $$\{\begin{array}{l} y_{\mathrm{rec}}(k)=a_{\mathrm{rec}}x(k)+b_{\mathrm{rec}}y_{\mathrm{rec}}(k-1)-c_{\mathrm{rec}}y_{\mathrm{rec}}(k-2)\\ \;\;\;\;\;\;\;\;\;\;\;\;\;\;\;\;\;\;+n_{\mathrm{rec}}(k)\\ a_{\mathrm{rec}}+b_{\mathrm{rec}}-c_{\mathrm{rec}}=1 \end{array}$$

where *n_res_*(*k*) and *n_rec_*(*k*) are the Gaussian noise added in the sensor response and recovery phases, respectively. In our simulations, $n_{\mathrm{res}}(k)∼N\left(0,\sigma_{\mathrm{res}}^{2}\right)$ and $n_{\mathrm{rec}}(k)∼N\left(0,\sigma_{\mathrm{rec}}^{2}\right)$, where σ*_res_* = σ*_rec_* = 5 ppm. The average response time of the TGS-series MOS sensors is about 1.8 s, and the average recovery time is 20.7 s without a fan and 11.1 s with a fan [39]. In our simulations, the response and recovery times are set as 2 s and 11 s, respectively.

### 6.4. Simulation Results

The size of the simulation environment is 100 m × 100 m. Each square grid of the environment is 0.5 m × 0.5 m. The rate of puff released by the source is 5 puffs/s. The plume-model update period is 0.01 s. The wind speed range is between 0.5 and 2.5 m/s. The gas source is located at (20, 0) and the robots start at (90, −30), where the coordinate units are meters. The gas-source localization algorithm is demonstrated for two different plume environments, which we refer to as slightly wandering and medium-wandering. The extents of the two plumes in the vertical direction are 20, 60 (measured at *x* = 100 m), respectively.

The CPSO (see Reference [27]) and P-PSO algorithms proposed in this paper are adopted by multi-robot for searching with the robot numbers 1, 3, 5, 7, 9, 11, 13 and 15. Each method was implemented 20 times for each of the slightly wandering, medium-wandering environments. The parameters for the CPSO were the same as those used in [27]. The time consumed by the robots for traveling from the start points to the vicinity of the gas source is considered as the performance metric. If none of the robots approached the gas source within 3000 s, the trial was taken as failure.

**Figure 6.**
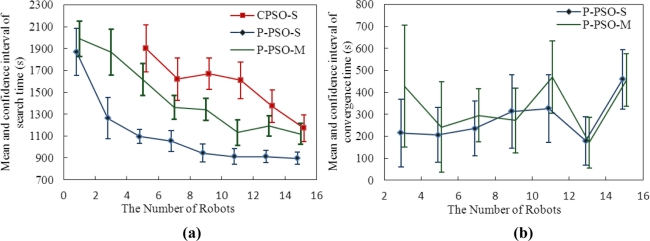
**(a)** The search time of P-PSO and CPSO based gas source localization in large-scale plume environments; **(b)** The convergence time of P-PSO.

The simulation results are illustrated in Figure 6, in which the abscissa is the number of robots and the vertical ordinate expresses the confidence interval of a search time with a 95% confidence level. The source declaration is not studied in this paper, so the search time in Figure 6(a) denotes that any robot enters into a circle *O*1 with a radius of *R*_1_ (*R*_1_ = 0.5 m) and the center being actual gas source. To reduce the chance of random arrival, a more rigorous metric, *i.e*., convergence time was used. The convergence time means the time from any robot entering into *O*1 to all the robots converging in a circle *O*2 with a radius of *R*_2_ (*R*_2_ = 5 m) and the center being actual gas source. Figure 6(b) shows the simulation results of convergence time. It is taken as a success search if any robot enters the circle *O*1, and the success times is shown in Table 1. In Figure 6 and Table 1, P-PSO-S and P-PSO-M indicate search results using the P-PSO-based method for the slightly wandering and medium-wandering plume environments, respectively. CPSO-S refers to the search results using the CPSO-based method for the slightly wandering plume environment. Within 3,000 s, few robots using the CPSO-based method for the medium-wandering plume environment (CPSO-M) approached the gas source, so their results are not shown in Figure 6. In addition, using one robot does not make sense for the CPSO algorithm, so in Figure 6 the searching time using one robot is not given, either.

It takes more time to localize the gas source via the P-PSO algorithm in medium-wandering plume environments than that in the slightly wandering environments. The trails adopting CPSO method in slightly meandering plume environments consume longer time than both the conditions employing P-PSO algorithms. That is, the P-PSO algorithm gains an advantage over the CPSO in respect of the searching efficiency. Furthermore, the searching time gets reduced for both algorithms as the number of robots increases. In contrast with the searching time, as Figure 6(b) shows, the convergence time in P-PSO trials increases with the increase in the number of robots. This phenomenon might be due to two reasons. Firstly, in the upwind region of the source, there is no gas plume, and in the downwind region of the source, the plume width is very narrow. Therefore, in the vicinity of the source, the chance of detecting plume is low and the possibility of missing the plume is high. Secondly, the algorithm of odor source declaration was not adopted in our research, so the robots which had approached the source continued searching and might move away from the source. Only all the robots approached the source simultaneously, was it taken as convergence. Thus, the larger the number of robots is, the harder to converge to the real gas source.

Table 1 shows the success times of P-PSO and CPSO algorithms. From Table 1 it is seen that the P-PSO algorithm manages to navigate the robots to approach the gas source at least 12 times (when only one robot was used) out of 20 trials. In the slightly meandering plume environments, the success times of CPSO obviously increases with an increase in the number of robots; in the medium-wandering plume environments, however, the success times of CPSO is very low on the whole, although it also increases as number of robots increases.

**Figure 7.**
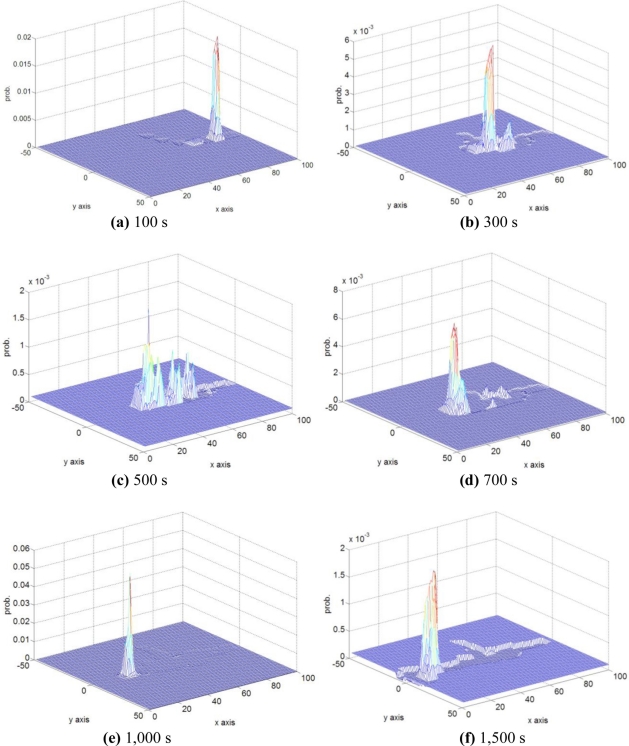
The combined gas source probability maps estimated at six different times.

Figure 7 shows the combined gas source probability maps derived from separate maps of 5 robots, which are recorded at six different times in one trail in the medium-wandering plume. The estimated highest probability peaks move from the start area to the location of the gas source during the robots’ searching process. In Figure 7(e), one of the robots approaches the gas source for the first time. The robots continue to search, and finally converge to the gas source, as shown in Figure 7(f).

## 7. Real Robot Experiments

The collective odor source estimation and search experiments were carried out in a centralized way. The sensed gas concentrations and airflow information were sent from each robot to a central workstation, and the control commands were sent from the workstation to each robot, both via wireless communication. If all the robots approach the real source and converge in a specified area, the algorithm is stopped manually.

### 7.1. Real-Robot Hardware Platform

**Figure 8.**
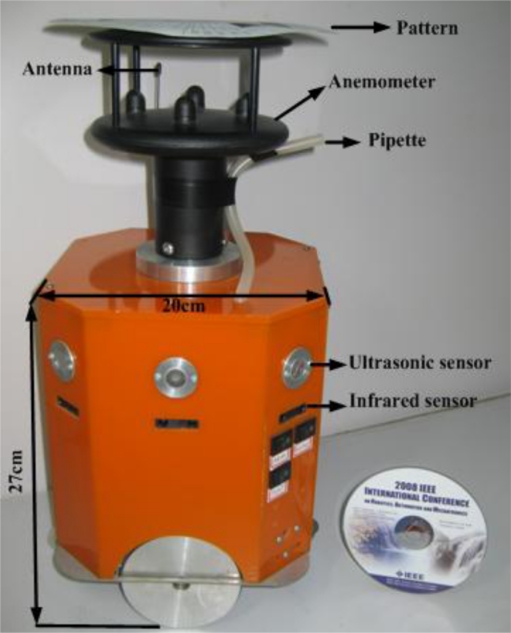
The small mobile MrCollie robot and onboard sensors.

Four small olfaction robots, named MrCollie (Mobile Robots for Cooperative Odor-source LocaLization in Indoor Environments), were used in the experiments. The robots were designed and assembled by the Institute of Robotics and Autonomous Systems of Tianjin University in 2006. One of the MrCollie robots and its onboard sensors is illustrated in Figure 8. The robot is driven differentially by two wheels, one mounted on the left and the other one on the right. Two castors on the front and back sides are used for balance. The robot is equipped with a two-dimension ultrasonic anemometer (Windsonic, Gill), a gas sensor (TGS2620, Figaro, with a response time of 1.4 s and a recovery time of 15.0 s), eight sonar sensors (L Series 40LPT16, Senscomp), eight infrared sensors (GP2D15, Sharp), and a wireless communication module (RPC module, Radiometrix). There is a unique location identifier at the top of each anemometer.

### 7.2. Gas Sensor

High sensitivity, long life-span and low cost make MOS sensors the most widely used gas sensors in mobile robots. TGS2620, a kind of MOS sensor produced by Figaro Engineering Inc., was used in our real-robot OSL experiments. TGS2620 consists of a silicon semiconductor layer formed on an alumina substrate of a sensing chip together with an integrated heater. In the presence of a detectable gas, the voltage across the heater causes an oxygen exchange between the volatile gas molecules and the metal coating material. Electrons are attracted to the loaded oxygen and result in decreases in sensor conductivity. A simple electrical circuit can convert the change in conductivity to an output signal which corresponds to the gas concentration [1,8].

The relationship between the gas concentration and the sensor resistance is expressed as follows [20]:

(27) $$R_{s}=R_{0}{\left(1+a\cdot C\right)}^{-b}$$

where *R_s_* and *R*_0_ represent the sensor resistances in gas and air, respectively; *C* means the gas concentration; *a* and *b* are constants. A signal processing circuit converts the change in resistance to output voltage *V_out_*:

(28) $$V_{\mathrm{out}}=V_{0}{\left(1+a\cdot C\right)}^{b}$$

where *V_0_* is the output voltage when *C* = 0.

**Figure 9.**
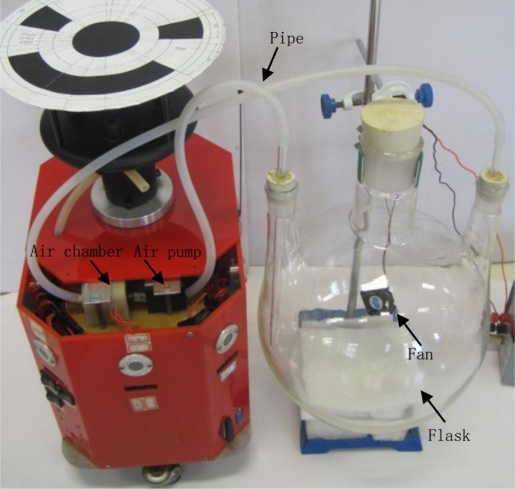
The device for gas sensor calibration. A TGS2620 gas sensor was mounted inside the air chamber.

The calibration process is described as follows: a certain amount of liquid ethanol was injected into a flask, and a fan was employed to speed up the evaporation. The amount of ethanol liquid was calculated according to the desired concentration of the ethanol vapor and the volume of the flask. The vapor was sucked by an air pump into a chamber and contacted with the gas sensor therein. The sensor outputs were recorded after the readings got steady. The calibration device is given in Figure 9. Through curve fitting, the constants *a* and *b* in Equation (28) could be obtained.

### 7.3. Robot Localization

An overhead charge coupled device (CCD) camera sent the image of each robot’s location identifier to the workstation, and the position and orientation of each robot were extracted by the workstation via a simple pattern recognition algorithm.

**Figure 10.**
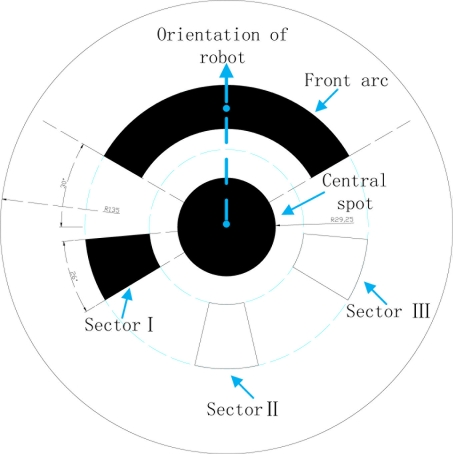
The location identifier labeled at the top of the robot.

The location identifier is shown in Figure 10. The central black spot indicates the position of the robot, and the straight line joining the spot and the front circular arc (120°) determines the orientation. In addition, the three sectors (Sectors I, II and III) beneath the central spot are used to distinguish the serial number of the robot. If a sector is filled with black, it represents 1; else, it represents 0. Thus, up to seven serial numbers (001∼111) can be distinguished. The white margin surrounding the black areas reduces the chance of adhesion to the cluttered background and the robot body.

The experimental scene is captured by the overhead CCD camera and sent to the workstation. Then the workstation can recognize the location identifier by a series of binarization, filtering and pattern recognition process. Finally, the position, orientation and serial numbers of the robots are obtained. Sometimes the CCD camera failed to localize the robots, so dead reckoning was also used for correction.

### 7.4. Obstacle Avoidance between Robots

**Figure 11.**
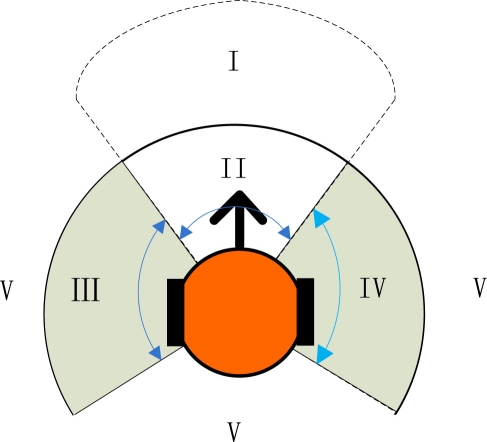
The surrounding area division for collision avoidance.

A traffic-rule based method was adopted to avoid robot collisions. The multiple robots are coordinated by seven simple rules. To apply these rules, the surrounding area of each robot is divided into five zones, see Figure 11. The bold arrow represents the orientation of the robot. Zone II stands for the immediate front space of the robot, while zone I the farther ahead. Zones III and IV indicate the left and the right space, respectively. The rest area belongs to the zone V. Each robot only responds to the nearest robot. For robot *i*, the nearest obstacle detected is assumed to be robot *j*, and the vectors from their current positions to their target points are denoted by *r⃗*_*i*_ and *r⃗*_*j*_, respectively.

The traffic rules applied by robot *i* are listed below:

1. If the nearest robot *j* appears in zone I, robot *i* turns right;
2. If robot *j* appears in zone II, robot *i* stops;
3. If robot *j* appears in zone III, and |*r⃗*_*i*_×*r⃗*_*j*_| > 0, then robot *i* moves right;
4. If robot *j* appears in zone III, and |*r⃗*_*i*_×*r⃗*_*j*_| < 0, then robot *i* turns left;
5. If robot *j* appears in zone IV, and |*r⃗*_*i*_×*r⃗*_*j*_| > 0, then robot *i* moves left;
6. If robot *j* appears in zone IV, and |*r⃗*_*i*_×*r⃗*_*j*_| < 0, then robot *i* turns right;
7. If robot *j* appears in zone V, then robot *i* does not respond.

The multi-robot system can realize basic collision avoiding functions by applying the above traffic rules, but the radii and angles of the five zones need to be adjusted in advance.

### 7.5. Experiment Arena

**Figure 12.**
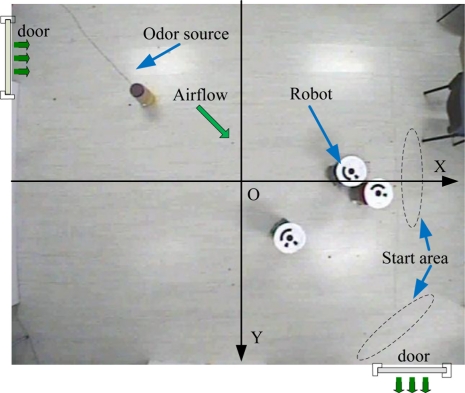
Real-robot experiment arena as seen from the overhead camera.

Figure 12 shows the experiment setting and coordinate framework. A humidifier filled with liquid ethanol was used as the odor source. The release rate was 25.35 mg/s. It was placed in the vicinity of the upper left door from which the wind came. The resultant odor plume spread diagonally from the odor source in the upper left corner to the opposite corner where the wind blew out through the other door.

**Figure 13.**
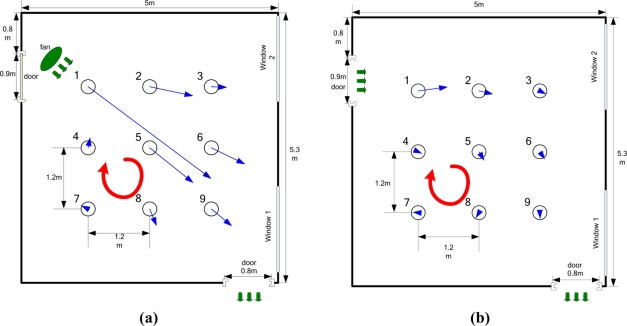
The average wind speed and direction of each anemometer over 300 s. **(a)** Artificial wind; **(b)** Natural wind.

Multi-robot CPT experiments were conducted in the laboratory of the Institute of Robotics and Autonomous System at Tianjin University. The laboratory had two doors and two windows. The area of the lab was 5.3 m × 5.0 m (the detailed dimensions can be seen in Figure 13). There were computer desks and chairs along the four sides of the laboratory. An overhead CCD video camera (3.6 m off the ground) was used to localize the robots and record the experiment processes. The coverage area of the CCD camera was about 4.8 m × 4.8 m.

### 7.6. Airflow Field Measurement

The robots moved in two different airflow fields, *i.e*., the artificial wind produced by an electric fan placed 1.5 m from the source and natural wind that blew when the two doors were open (the windows were closed). Before the gas-source localization experiments, the two airflow fields were measured and analyzed using nine two-dimension ultrasonic anemometers (Windsonic, Gill). The average wind speed and direction measured in the artificial and natural airflow fields by anemometer over 300 s are shown in Figure 13, where the length and direction of each blue arrow represent the average wind speed and direction, respectively.

As Figure 13 shows, the flow directions in the lower-left corner of artificial and natural flow fields both changed greatly because of the indoor boundary, so that a big and stable eddy was formed (see Figure 13, the red circle with arrow). The flow fields were not homogeneous in the big eddy region or the room boundary, the assumption of estimating the separate gas source probability presented in Section 2 does not fit here. In other region of the room, the flow directions had little change in a small area, so we could consider the flow fields in other regions as approximate homogeneous.

### 7.7. Experiment Results

In the experiments, the robots moved in a run-stop-run-stop mode (running for 5 s and stopping for 5 s). The motion speed of each robot was set to 2.5 cm/s–∼4 cm/s. Both the airflow and gas concentration were sampled five times during the 5-second-stop.

The robots searched in two different indoor environments, one is artificial airflow, and the other is natural airflow. For each airflow environment, the robots started from the right side and the lower right corner of the search area. Thus, there are four different experimental situations. The gas source localization experiments were run 40 times in total, 10 trials for each situation. Before each new experiment was run, the doors and windows were opened till the detected gas concentration was less than 5 ppm. If the three robots did not approach the gas source (*i.e*., did not come within 50 cm) within 15 min, it was thought the gas source localization processes failed.

**Figure 14.**
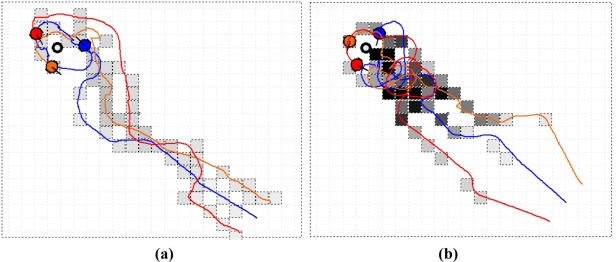
Two of recorded collective OSL and search processes, the robots started from the lower right corner. **(a)** Artificial wind (the total search time was 413 s); **(b)** Natural wind (the total search time was 862 s).

Figure 14 presents two of the collective OSL and search processes when the three robots started from the lower right corner. The grids by which the robots passed and in which the measured concentration was higher than a threshold (the initial value was 50 ppm, and was increased in proportion with the maximum concentration) were marked with dashed lines, with darker lines indicating higher concentrations. The red, blue and orange curves indicate the trajectories of the three robots, respectively. To find a plume, the robots moved toward three different directions (30° between adjacent directions) at the beginning. The initial coordinates of the blue robot were (200, 200), with centimeters as the coordinate units. When the gas concentration detected by any robot was higher than 50 ppm, the gas plume was thought to be found and the robots moved on the basis of the proposed estimation and searching algorithm. When the distance between each robot and the gas source was less than 50 cm, the searching was stopped manually. The tracing trajectories in natural wind [Figure 14(b)] are more tortuous than that in artificial wind [Figure 14(a)], indicating that it takes more time to search in natural wind environment. That is, it is more difficult to localize the odor source in natural airflow field.

The experimental results are presented in Table 2, in which “RS” and “LR” indicate starting at the right side and lower right corner, respectively; and “AW” and “NW” indicate the artificial and natural winds, respectively. The performance is evaluated by *T_av_* and *R_a_*, where *T_av_* means the average search time of 10 trials, and *R_a_* indicates the success times of approaching the source out of 10 trials. Here it should be noted that in the real-robot experiments, the success means that all the three robots converge near the source, and the search time is calculated from the beginning of one trial to all the three robots converge near the source.

From the experimental results it can be found that the average search time for the artificial wind fields is shorter than that for the natural wind fields. By analyzing the experiment processes and results, we think this is due to at least two reasons. First, the variation in the direction of the natural wind was greater than that of the artificial wind. Second, in the natural wind field, sometimes there existed long-duration weak airflows (less than 5 cm/s, which the anemometer could not detect reliably). The reasons that the search time from the right side was shorter than that from the lower right corner might be explained from two aspects. First, the distance between the robots and the real gas source is shorter for the right-side starting location. Second, the robots starting from the lower right corner were apt to fall into the big eddy field, which resulted in useless search for a period of time, so the total time increased.

As mentioned above, the big eddy area (see the red circle in Figure 13) is one of main reasons that caused long search times or even search failures. The gas molecules accumulated and a local concentration maximum was formed in the big eddy area. To keep the robots from falling into this area, the detection threshold of sensor was set to be increased in proportion with the maximum concentration during the searching process. In addition, if the posterior probability of gas source estimated by other robots was higher than that estimated by the robot fell into the big eddy area, or the threshold of gas sensor in that time is already higher than the concentration detected in the big eddy area, the robot could escape from the eddy area and finally the gas source could be found; otherwise, all the robots could entered the big eddy area and in the limited time period all the robots could not escape. Anyway, the multi-robot search, instead of single robot, combining the gas source estimation strategy could increase the robustness of the system and reduce the probability of falling into local area.

Although one run failed owing to the big eddy, the other nineteen experiment runs for the artificial airflow field succeeded. For the natural airflow case, two failures (one for RS-NW and one for LR-NW) were because of long-duration weak airflow (less than 5 cm/s, which the anemometer could not detect reliably), one failure (for LR-NW) was due to the big eddy.

## 8. Conclusions

Simulation results using time-varying and large-scale advection–diffusion plume models demonstrate the feasibility and robustness of the proposed odor source localization method via multi-robot search and estimation. Compared with the CPSO based method, the plume-tracking strategy based on the estimation-searching frame proposed in this paper can find the single odor source in less time with a higher success rate. For slow-changing airflow environments (slightly wandering large-scale advection–diffusion plumes, for example), relatively few robots using the proposed plume-tracking strategy can successfully approach the odor source, and the use of more robots does not noticeably decrease the search time. It takes longer to search in the medium-wandering plume environment using the P-PSO-based method. Therefore, the P-PSO-based plume-tracking method has good robustness regarding different plume environments when the number of robots is sufficient. The proposed multiple-robot based collective gas-source localization method is also demonstrated with real robots experiments in indoor time-variant airflow environments. Except the extreme airflow conditions such as the long-period weak airflow and big eddy areas, the proposed method works well in both the natural and artificial airflow fields. Limited by our experimental infrastructure, the proposed OSL strategy was only evaluated in a small-scale indoor environment. If the infrastructure is improved, the strategy might be extended to large-scale scenarios, even outdoor airflow environments.

The feasibility and robustness of the proposed multi-robot gas source localization method comes from two aspects. First, the tradeoff between exploration and exploitation is achieved in the proposed gas source localization strategy. The estimation process exploits the detected information to guide multi-robot search, while the multi-robot search updates the estimation result by exploring more areas. Second, the gas source probability estimated using both the gas and airflow information, instead of simple gas concentration and wind direction, is utilized.

The proposed P-PSO method might fail in the region where obstacles or boundaries (e.g., wall) exist because the hypotheses of homogeneity and isotropy are false. How to estimate gas source probability in such situations will be our next research.

## Figures and Tables

**Table 1.** The times of robots successfully approaching the source out of 20 trails.

**Number of robots**	**1**	**3**	**5**	**7**	**9**	**11**	**13**	**15**
P-PSO-S	15	17	19	20	20	20	20	20
CPSO-S	—	2	5	12	13	17	17	19
P-PSO-M	12	16	18	18	19	20	20	20
CPSO-M	—	0	0	1	1	2	2	4

**Table 2.** Experimental results for plume finding/tracking experiments.

	**RS-AW**	**RS-NW**	**LR-AW**	**LR-NW**
*T_av_*(s)	309	493	521	709
*R_a_*	10	9	9	8

## Notations

*z*: The detection of wind and gas event
*z_t_*: The detection event z happening in the time period [*t*, *t* + *λt*]
*z̄_t_*: Undetected event happening in the time period [*t*, *t* + *λt*]
*z*_*t,In*_: Detected event, the robot is in the plume
$\bar{z_{t,\mathrm{Edge}}}$: Undetected event, the robot is on the edge of the plume
$\bar{z_{t,\mathrm{Out}}}$: Undetected event, the robot is outside of the plume
*z*_*i,t*_: Detection event by the robot i in the time period [*t*, *t* + *λt*]
*Z_t_* = {*z*_1*,t*_, *z*_2*,t*_, …, *z*_*N,t*_}: Detection vector by N robots in the time period [*t*, *t* + *λt*]
*Z*_1_*_t_* = {*Z*_1_,*Z*_2_, …, *Z*_*t*_}: Detection vector by N robots from time 1 to t
Δ: A small connected domain
*m*: The smallest space unit of gas source probability estimation
*m_xy_*: The grid with the central coordinate (*x,y*) in global coordinate system
*m*_*x′,y′*_: The grid with the central coordinate (*x′,y′*) in local coordinate system
*p*(*Δ*): Probability that the gas source is in Δ
*p*(*m*): Posterior probability that the gas source is in *m*
*p*(*m*|*z*): Posterior probability that the gas source is in *m* when *z* happens
*p*(Δ|*z*): Posterior probability that the gas source is in Δ when *z* happens
*p*(*m*|*z*,Δ): Posterior probability that the gas source is in *m* when *z* happens and the gas source is in Δ
*p_l_*(*m*′_*x′y′*_): (Prior probability that the gas source is in the grid *m′*_*x′y′*_ in local coordinate system
*p_l_*(*z*_*t,In*_|*m*′_*x′y′*_): Conditional probability that *z_t,In_* happens given the gas source is in *m*′_*x′y′*_ in local coordinate system
*p_l_*(*z̄*_*t*_|*m*′_*x′y′*_): Conditional probability that *z̄_t_* happens given the gas source is in *m*′_*x′y′*_ in local coordinate system
*p_l_*(*m′*_*x′y′*_|*z*_*t*_,Δ): Posterior probability of any grid *m′*_*x′y′*_ being gas source when the source is in the area Δ and *z_t_* happens in local coordinate system
*p_l_*(*m′*_*x′y′*_|*z*_*t,In*_,Δ): Posterior probability of any grid *m′*_*x′y′*_ being gas source when the source is in the area Δ and *z_t,In_* happens in local coordinate system
*p_l_*(*m′*_*x′y′*_|*z̄*_*t*_,Δ): Posterior probability of any grid *m′*_*x′y′*_ being gas source when the source is in the area Δ and *z̄_t_* happens in local coordinate system
*p_l_*(*m′*_*x′y′*_|*z*_*t,In*_): Posterior probability of any grid *m′*_*x′y′*_ being gas source when *z_t,In_* happens in local coordinate system
$p_{l}\left({m^{\prime }}_{x^{\prime }y^{\prime }}|\bar{z_{t,\mathrm{Edge}}}\right)$: Posterior probability of any grid *m′*_*x′y′*_ being gas source when $\bar{z_{t,\mathrm{Edge}}}$ happens in local coordinate system
$p_{l}\left({m^{\prime }}_{x^{\prime }y^{\prime }}|\bar{z_{t,\mathrm{Out}}}\right)$: Posterior probability of any grid *m′*_*x′y′*_ being gas source when $\bar{z_{t,\mathrm{Out}}}$ happens in local coordinate system
*p_l_*(Δ|*z*_*t*_): Posterior probability of the gas source being in the area Δ when *z_t_* happens in local coordinate system
*p_l_*(Δ|*z*_*t,In*_): Posterior probability of the gas source being in the area Δ when *z_t,In_* happens in local coordinate system
$p_{l}\left(\Delta |\bar{z_{t,\mathrm{Edge}}}\right)$: Posterior probability of the gas source being in the area Δ when $\bar{z_{t,\mathrm{Edge}}}$ happens in local coordinate system
$p_{l}\left(\Delta |\bar{z_{t,\mathrm{Out}}}\right)$: Posterior probability of the gas source being in the area Δ when $\bar{z_{t,\mathrm{Out}}}$ happens in local coordinate system
*p*(*m*_*xy*_|*z*_*i,t*_): Separate probability of the gas source being in *m_xy_* by the detection event of the robot i at the time t within the small area Δ
*p*(*m*_*xy*_|*z*_*t*_): Combined probability of the gas source being in *m_xy_* by the detection event of all the robots at the time t
*p*(*m*_*xy*_|*z*_1*t*_): Combined probability of the gas source being in *m_xy_* by the detection event of all the robots from time 1 to t
